# Evaluation of the Effect of Aegle marmelos (Bael Leaf) Extract on Human Fibroblast Viability: An In Vitro Study

**DOI:** 10.7759/cureus.72466

**Published:** 2024-10-27

**Authors:** Mandip Dey, Sunanda Rao, Ravishankar PL, Prem Blaisie Rajula, Gayathri K, Murali Venkata Rama Mohan Kodali

**Affiliations:** 1 Periodontology, SRM Kattankulathur Dental College and Hospital, SRM Institute of Science and Technology, Chengalpattu, IND; 2 Oral and Maxillofacial Surgery, King Faisal University, Al-Hofuf, SAU

**Keywords:** aegle marmelos, cytotoxicity, ethanolic extract, fibroblasts, plant extracts

## Abstract

Aim

To evaluate the biocompatibility of ethanolic extract of *Aegle marmelos* (bael/vilvam) leaves with gingival fibroblast cells.

Materials and methods

Commercially available bael leaves were used to produce a herbal ethanolic extract using the cold percolation technique. Evaluation of cytotoxicity of the bael leaf extract (BLE) on fibroblast cell line at six different concentrations (200µl/ml, 100µl/ml, 50µl/ml, 25µl/ml, 12.5µl/ml, 6.3µl/ml) was done using MTT assay. The concentration with most cell viability was then evaluated at different time points (one, two, four, six, 10 minutes) for viability in the same cell line.

Results

The results showed that the 6.3 µl/ml had the least cytotoxic effect on fibroblasts. The cell viability decreased as exposure to the extract increased (one minute to 10 minutes).

Conclusion

The ethanolic extract of *Aegle marmelos* (bael) leaves exhibited minimal cytotoxicity at a concentration of 6.3 µl/ml on gingival fibroblasts, with cell viability decreasing as exposure time increased.

## Introduction

A complex interplay between periodontopathogens, their by-products, and the host response is the cause of periodontal disease, a persistent immuno-inflammatory disorder. It is marked by the disintegration of the periodontal ligament and alveolar bone, leading to clinical attachment loss. Effective plaque control is essential for preventing this chronic disease. Chlorhexidine (CHX) is widely regarded as the exemplary benchmark for periodontal therapy owing to its broad-spectrum antimicrobial properties and efficacy [1-3]. Since its introduction in the 1950s, CHX has become an integral therapeutic agent in managing periodontal diseases and maintaining oral hygiene. Its use is associated with significant reductions in plaque and gingival indices.

However, evidence suggests that CHX may have deleterious effects on gingival fibroblast proliferation, gingival epithelial cells, periodontal ligament cells, cultured alveolar bone cells, and osteoblastic cells [4-6]. Other side effects of CHX include brownish discoloration and an unpleasant taste. Given the potential adverse effects associated with CHX, exploring alternative herbal-based mouthwashes with similar antimicrobial properties but fewer side effects is warranted.

Herbal medicine has a rich history and remains a crucial component of healthcare globally. The World Health Organization (WHO) estimates that approximately four billion people, or 80% of the global population, rely on herbal remedies for primary healthcare. In India, the Botanical Survey reports over 8,000 species of medicinal plants, which have been used since ancient times to treat various systemic and dental conditions [7]. The exploration of medicinal plants for novel beneficial compounds is critical due to their safety profile and minimal adverse effects. Bael (*Aegle marmelos*) offers a promising solution.

*Aegle marmelos*, an herb used in traditional Indian medicine, belongs to the Rutaceae family. The leaves have numerous ethnobotanical applications. A paste from these leaves effectively treats ulcers; juice from young leaves cures eye infections, ophthalmia, and asthma, and acts as a laxative [7]. A decoction of the leaves serves as an expectorant and febrifuge.

The leaves, roots, and fruits of *Aegle marmelos* possess antimicrobial properties that can combat a range of bacterial types [7-9]. Limonene, the primary constituent of bael leaf extract, offers therapeutic properties such as analgesic, anti-inflammatory, anti-bacterial, anti-pyretic, and wound healing [7-9]. Essential oil from the leaves shows action against Aeromonas species and *Escherichia coli*, while the root's ethanolic extract demonstrates antimicrobial activity against *Vibrio cholerae, Salmonella typhimurium, and Staphylococcus aureus*. Leaf extracts have proven effective against *Escherichia coli*, enhancing bael's value in traditional medicine [7].

This study aims to evaluate the biocompatibility of bael leaf extract (BLE) with gingival fibroblasts, exploring its potential as a safer alternative for periodontal therapy.

## Materials and methods

The ethical clearance for the research design was granted by the Institutional Review Board (Ref No. SRMIEC-ST0324-976), and the study was conducted at the Department of Periodontology, SRM Kattankulathur Dental College and Hospital, Chengalpattu, Tamil Nadu.

Plant material and preparation

Commercially available *Aegle marmelos* (bael) leaves were obtained, dried in a hot air oven at 55°C for 12 hours, and subsequently ground into a fine powder. The extraction was performed using a 70% ethanol solution via cold percolation over 48 hours. In this process, 50 grams of powdered bael leaves were immersed in 150 ml of ethanol. After the 48-hour period, the extract was collected and subjected to evaporation using a vacuum evaporator to achieve a constant weight. The final extract was then stored at 4°C for further analysis.

Cytotoxicity evaluation

The 3-(4,5-dimethylthiazol-2-yl)-2,5-diphenyltetrazolium bromide (MTT) assay was used to assess the cytotoxicity. Fibroblast cells were cultivated in Dulbecco's Modified Eagle Medium (DMEM) enriched with 10% fetal bovine serum (FBS) at a seeding density of 5x10^4 cells per well in a 96-well flat-bottom microplate. Upon achieving sufficient confluence, the growing medium was eliminated. The cells were preserved overnight at 37°C in an atmosphere of 95% humidity and 5% CO2. Different concentrations of bael leaf extract (BLE) (200 µl/ml, 100 µl/ml, 50 µl/ml, 25 µl/ml, 12.5 µl/ml, and 6.3 µl/ml) were then added, and the cells were incubated for the following 48 hours. Subsequently, the wells were washed twice with phosphate-buffered saline (PBS), after which 20 µL of MTT solution was added to each well (MTT undergoes reduction to formazan within viable cells). The plate was incubated at 37°C, and after four hours, 100 µL of dimethyl sulfoxide (DMSO) was introduced to solubilize the formazan crystals. This absorbance was quantified with a microplate reader at 570 nm. The amount of light absorbed is proportional to the amount of formazan accumulated inside and on the cell surface. The deeper the purple color, the higher the absorbance, indicating a higher proportion of living cells. 

For the time-course experiment, cells were incubated for varying durations (one, two, four, six, eight, and 10 minutes) at a constant concentration of 6.3 µl/ml BLE. The MTT assay was similarly implemented to assess the viability of the cells.

An inverted phase-contrast microscope (Olympus CKX41 (Olympus Corporation, Japan) coupled with an Optika Pro5 CCD Camera (OPTIKA S.r.l., Italy)) was used to observe the entire plate after 48 hours of incubation of fibroblast cells with varying concentrations of BLE (200 µl/ml, 100 µl/ml, 50 µl/ml, 25 µl/ml, 12.5 µl/ml, and 6.3 µl/ml). Microscopic images were similarly obtained for various time intervals at 6.3 µl/ml concentration. Potential indicators of cytotoxicity were identified in the form of visible changes in cell morphology.

## Results

To evaluate the cytotoxicity of bael leaf extract on gingival fibroblasts, an MTT assay was performed following 48 hours of incubation with six different concentrations (200 µl/ml, 100 µl/ml, 50 µl/ml, 25 µl/ml, 12.5 µl/ml, 6.3 µl/ml). A dose-dependent decrease in cell viability was observed, with the highest cell viability at 6.3 µl/ml (95.5 ± 1.5%) and the lowest at 200 µl/ml (9.5 ± 0.2%). The IC50 value was determined to be 11.03 ± 0.1 µg/ml (Table 1).

**Table 1:** Mean percentage of fibroblast cell viability at different concentrations of BLE. BLE: bael leaf extract

Sample	Concentration µl/ml	Cells Viability (%)			Mean±SD (%)
Bael leaf extract ( BLE)	200.0	9.1	9.1	9.5	9.5±0.2
	100.0	10.0	9.9	9.9	9.9±0.05
	50.0	12.6	10.9	11.5	11.3±0.8
	25.0	16.7	15.7	15.3	15.9±0.7
	12.5	32.1	34.1	33.0	33.4±0.6
	6.3	95.3	94.2	97.1	95.5±1.5
	Negative control	100			100
	IC_50_ (µg/ml)				11.03±0.10

**Figure 1:**
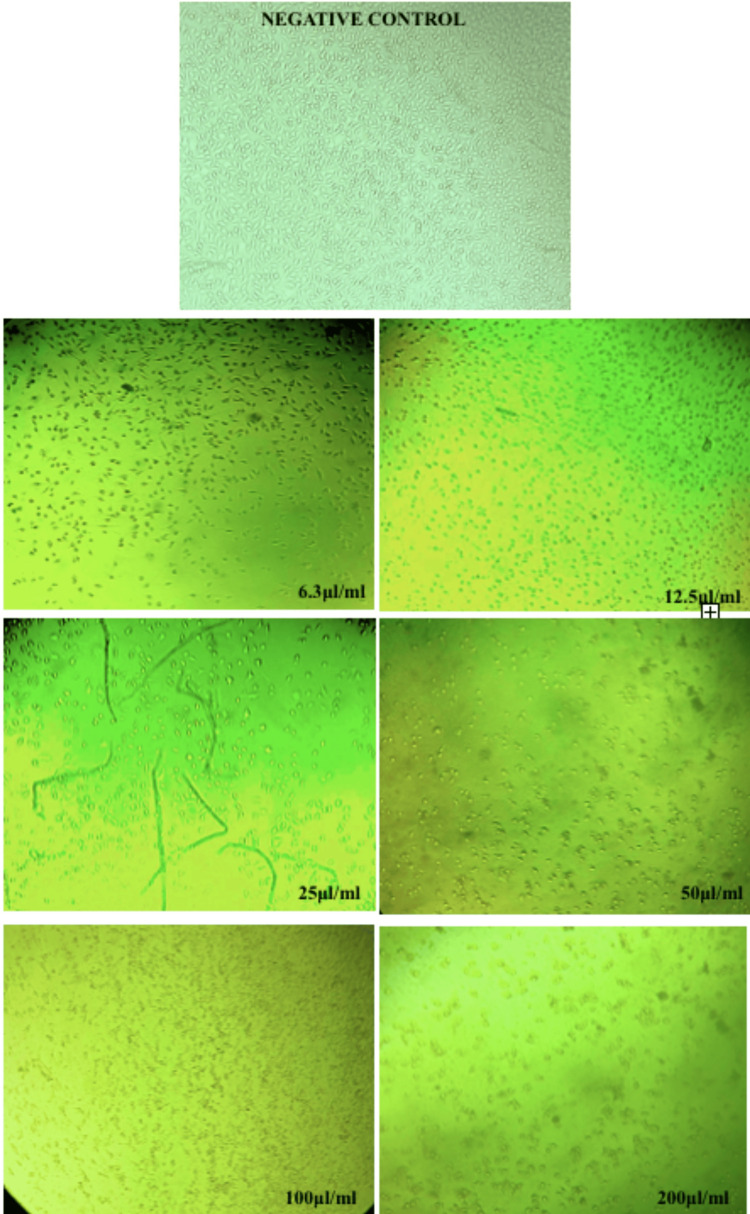
Inverted phase-contrast microscopic images of gingival fibroblasts subjected to various concentrations of BLE. BLE: bael leaf extract

The changes in the morphology of BLE-exposed fibroblasts were evaluated using phase-contrast microscopy. Increasing concentrations of BLE from 6.3 µl/ml to 200 µl/ml showed changes such as a decrease in cell density, nuclear fragmentation, and loss of cell morphology (Figure 1).

To assess the time-dependent effect, gingival fibroblasts were treated with 6.3 µl/ml BLE for different time intervals (one, two, four, six, eight, and 10 minutes). A slight decrease in cell viability was observed over time, with a mean viability of 100.04 ± 0.30% at one minute and 98.53 ± 0.99% at 10 minutes (Table 2).

**Table 2:** Mean concentration of fibroblast cell viability at a BLE concentration of 6.3µl/ml at different time periods. BLE: bael leaf extract

Sample	Time (mins)	Cells Viability (%)			Mean ± SD (%)
BLE (6.3µl/ml)	10	99.66	97.86	98.06	98.53±0.99
	8	97.92	98.78	99.48	98.73±0.78
	6	98.06	98.86	100.74	99.22±1.38
	4	98.92	99.30	100.88	99.70±1.04
	2	99.90	100.14	99.44	99.83±0.36
	1	100.28	100.14	99.70	100.04±0.30

**Figure 2:**
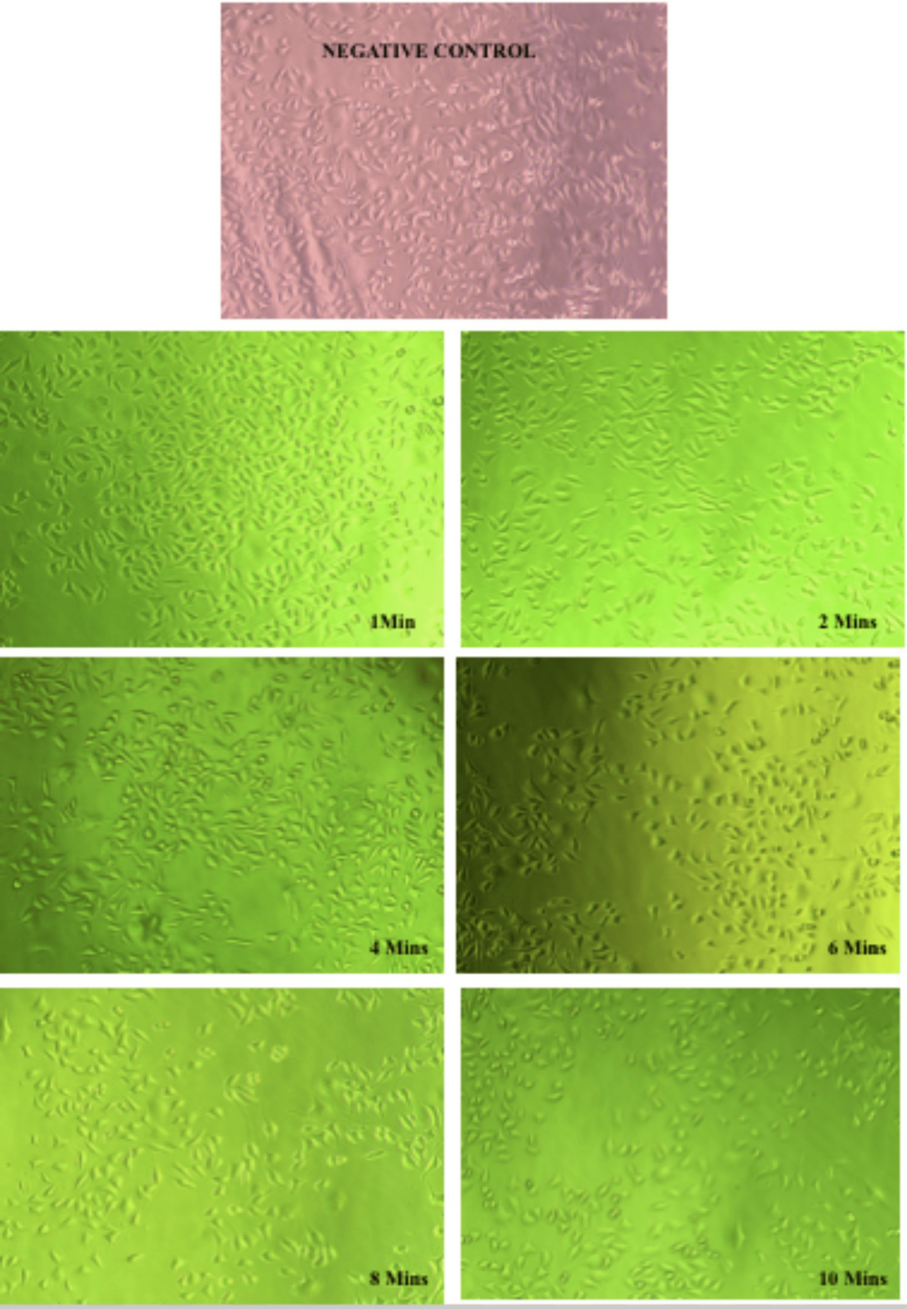
Inverted phase-contrast microscopic images of gingival fibroblasts subjected to 6.3 µl/ml concentration of BLE at various time intervals. BLE: bael leaf extract

At 6.3 µl/ml, fibroblasts predominantly exhibited normal morphology of cells. As the exposure time to BLE increased, minor alterations in the cellular morphology, such as loss of spindle shape of cells and cellular shrinkage, were seen (Figure 2).

## Discussion

Current periodontal therapy often uses chlorhexidine digluconate as an adjunct for plaque control. While effective, CHX raises concerns due to its potential toxicity towards fibroblasts, which are essential cells for maintaining healthy periodontal tissues [4-6]. Numerous studies have evaluated the effects of CHX on fibroblast viability. In 1992, Cline and Layman specifically investigated the viability of fibroblasts in response to increasing concentrations of CHX. Their findings demonstrated a significant reduction in fibroblast viability in a concentration-dependent manner, indicating that higher concentrations of CHX led to progressively greater cytotoxic effects [10]. Additionally, they observed that even at lower concentrations, CHX was toxic to various cell types, inclusive of gingival fibroblasts, highlighting its broad cytotoxic potential [11]. This highlights the need to identify safer alternatives.

The leaves, roots, and fruits of *Aegle marmelos* (bael) exhibit significant antimicrobial activity against a broad spectrum of bacterial species and anti-inflammatory effects due to the presence of various phytochemicals like skimmianine, marmelosin, and quercetin. Limonene, the predominant compound in bael leaf extract, has been shown to possess multiple therapeutic properties, including analgesic, anti-inflammatory, antibacterial, antipyretic, and wound-healing effects [5]. Given the key role of inflammation, microbial imbalance, and oxidative stress in the pathogenesis of periodontitis, these properties of bael leaves could potentially help to address the underlying drivers of this disease. Previous studies have evaluated the effects of *Aegle marmelos* leaf extract on various common oral bacteria and periodontal pathogens, demonstrating its potential efficacy [5,6]. The present study explored the biocompatibility of BLE with gingival fibroblasts by examining its cytotoxicity using the MTT assay.

The results revealed an IC50 value of 11.03 ± 0.1 µg/ml, suggesting moderate toxicity. Results showed a dose-dependent response, with lower concentrations of bael extract exhibiting reduced cytotoxicity towards fibroblasts; 6.3 µl/ml showed 95.5 ± 1.5% viability, while the 200 µl/ml concentration showed only 9.5 ± 0.2% viability. It was also observed that the mean viability of fibroblasts decreases as the time period increases (one, two, four, six, eight, and 10 min). At 6.3 µl/ml concentration of bael extract, the mean percentage of fibroblast viability varies from 100.04% to 98.53% for the time phase between one to 10 minutes. 

This cytotoxicity profile positions BLE as a potential candidate for future investigations to establish its efficacy as a biocompatible adjunct in plaque control. The established role of gingival fibroblasts in maintaining periodontal health through tissue homeostasis and wound healing necessitates the selection of antiplaque agents with minimal cytotoxicity towards these crucial cells. 

Prior studies have established the minimum inhibitory concentration (MIC) of BLE to be at 0.2%, corresponding to the 6.3 µl/ml concentration in this study, which further solidifies the findings of this study since the viability of the fibroblast cells is high at the concentration [7,11]. The results strongly support the hypothesis that BLE is less cytotoxic to gingival fibroblasts than CHX. Although the study did not directly compare BLE to CHX, the absence of cytotoxicity observed with BLE stands in stark contrast to the known cytotoxic effects of CHX.

While this study establishes the initial promise of BLE, future research should focus on the specific phytochemicals responsible for BLE's biological activities and evaluate its efficacy on other cell lines as well. Additionally, direct comparisons with CHX, the gold standard, and other antiplaque agents in terms of cytotoxicity would provide valuable insights into BLE's clinical potential.

We acknowledge the potential drawbacks of the study, including the use of a single cell line and the use of the entire extract rather than the isolated active compound.

## Conclusions

In conclusion, this study demonstrated the safety of *Aegle marmelos* extract on human gingival fibroblasts, underscoring its potential as a biocompatible agent for periodontal applications. Given this finding, BLE may serve as an effective and safe alternative in oral rinses and other plaque-controlling adjuncts in periodontal therapy. Further research should explore its long-term efficacy in managing bacterial populations while ensuring minimal adverse effects, positioning it as a promising addition to periodontal treatment regimens.
